# IL-9 plays a critical role in helminth-induced protection against COVID-19-related cytokine storms

**DOI:** 10.1128/mbio.01229-24

**Published:** 2024-06-20

**Authors:** ChunXiao Xiang, Gang Zhong, Huiqing Wang

**Affiliations:** 1 Medical Simulation Centre, West China Second University Hospital, Sichuan University, Chengdu, China; 2 University/Trauma Center, West China Hospital, Sichuan University, Chengdu, China; 3 Department of Pediatrics, West China Second University Hospital, Sichuan University, Chengdu, China; University of Calgary, Calgary, Canada

**Keywords:** IL-9, COVID-19, cytokine storms, *Trichinella sprialis*

## Abstract

A recent study published in *mBio* by Cao et al. demonstrated that the helminth *Trichinella sprialis* (Ts) alleviates COVID-19-related cytokine storms in an IL-9-dependent way (Z. Cao, J. Wang, X. Liu, Y. Liu, et al., mBio 15:e00905-24, 2024, https://doi.org/10.1128/mbio.00905-24). A cytokine storm is a severe immune response characterized by the overproduction of proinflammatory cytokines, such as TNF-α and IFN-γ, leading to tissue damage and mortality in COVID-19 patients. This study indicated that IL-9 is crucial in protecting against cytokine storm syndromes associated with SARS-CoV-2 infection and proposed that anti-inflammatory molecules from Ts excretory/secretory (TsES) products could be a novel source for treating such illnesses.

## COMMENTARY

The COVID-19 pandemic has created unprecedented concerns for world health (1, 2), with cytokine storm syndrome as a significant contributor to the severity and mortality of the disease (3). Hyperactivation of proinflammatory type 1 cytokines, including TNF-α and IFN-γ, is a characteristic of severe COVID-19 (4). These cytokines are crucial for mediating the immune system’s initial defense against viral infections. This response not only curbs the spread of the virus within the host but also activates other immune system components essential for viral clearance. In contrast, hyperactivation of these cytokines can cause what is known as a “cytokine storm.” This hyperinflammatory condition results in severe tissue damage and inflammation, mirroring the pathological changes observed in severe cases of COVID-19. Essentially, the cytokine storm constitutes an overly intense immune response that becomes detrimental and potentially fatal to the host (5). However, there is an urgent need for effective therapeutics against cytokine storms to treat lethal COVID-19.

Throughout human history, helminth infections have existed, and a sizable fraction of these parasitic illnesses are asymptomatic. The hygiene hypothesis suggests that advancements in sanitation and reduced pathogen exposure in developed nations have contributed to the increase in inflammatory disorders (6). This increase might be partially due to the reduced prevalence of helminth infections, which are known to modulate the immune system and reduce proinflammatory responses (7). Recent epidemiological research, particularly from regions in Africa, has indicated a correlation between helminth coinfection and milder COVID-19 symptoms (8). Recent evidence also points to a potential type 2 microenvironment linked to helminth infection that can improve the progression of SARS-CoV-2 infection (9). Helminths, such as *Trichinella spiralis* (Ts), are known to induce a strong type 2 immune response characterized by the activation of type 2 cells and the release of cytokines such as IL-4, IL-5, IL-9, and IL-13 (10). These insights could lead to novel therapeutic approaches that harness the immunomodulatory effects of helminth infections to treat or prevent inflammatory responses related to SARS-CoV-2 infection.

In a study published in *mBio*, Cao et al. investigated the connections between helminth-induced type 2 cytokines and proinflammatory cytokine storms, as well as their potential implications for viral infections such as COVID-19 (11). Investigators have shown that prior Ts infection can protect against cytokine shock induced by TNF-α and IFN-γ. This protective effect is dependent on IL-9 but not IL-4Rα (a common subunit receptor for IL-4 and IL-13). IL-9 alone can offer protection against the cytokine storm related to SARS-CoV-2 infection, suggesting a critical role for this cytokine in managing excessive inflammatory responses. This study revealed that Ts excretory/secretory (TsES) products were more effective than IL-9 treatment alone during SARS-CoV-2 infection. This finding suggests that these helminth-derived products may contain additional immunomodulatory components or work together to improve anti-inflammatory responses above and beyond what IL-9 alone may do. The efficacy of Ts-derived products in modulating immune responses opens the door to their use as biologics, namely biologically derived therapeutic products. Furthermore, understanding the mechanisms via which Ts controls immune responses can aid in the discovery of new therapeutic targets, potentially leading to the development of innovative therapies for inflammatory disorders and infections.

In conclusion, this study advances our understanding of the potential therapeutic applications of helminth-derived compounds. This study presents a compelling argument for the therapeutic potential of helminths and their derived products in managing cytokine storms, particularly in the context of COVID-19. These findings are significant because they offer a novel perspective on the modulation of immune responses during viral infections, which could have profound implications for the treatment of patients with severe COVID-19. However, the study also has limitations that warrant further investigation. The specific components of TsES that confer the observed protective effects remain unidentified, necessitating future research to isolate and characterize these molecules. Exploring the potential of helminth-derived therapies in other inflammatory and autoimmune conditions could offer new treatment avenues for a range of diseases, highlighting how natural parasitic infections can inform modern medical strategies and innovations.
